# An Acetylcholinesterase Antibody-Based Quartz Crystal Microbalance for the Rapid Identification of Spinal Ventral and Dorsal Roots

**DOI:** 10.1371/journal.pone.0069049

**Published:** 2013-07-23

**Authors:** Tao Sui, Yingbin Ge, Wujun Liu, Zongbao K. Zhao, Ning Zhang, Xiaojian Cao

**Affiliations:** 1 Department of Orthopedics, The First Affiliated Hospital of Nanjing Medical University, Nanjing, People’s Republic of China; 2 Department of Physiology, Nanjing Medical University, Nanjing, People’s Republic of China; 3 Division of Biotechnology, Dalian Institute of Chemical Physics, CAS, Dalian, People’s Republic of China; Weizmann Institute of Science, Israel

## Abstract

Differences in the levels of acetylcholinesterase (AChE) in ventral and dorsal spinal roots can be used to differentiate the spinal nerves. Although many methods are available to assay AChE, a rapid and sensitive method has not been previously developed. Here, we describe an antibody-based quartz crystal microbalance (QCM) assay and its application for the quantification of AChE in the solutions of ventral and dorsal spinal roots. The frequency variation of the QCM device corresponds to the level of AChE over a wide dynamic range (0.5–10 µg/ml), which is comparable to the response range of the ELISA method. The frequency shift caused by the ventral roots is 3-fold greater than that caused by the dorsal roots. The antibody-based QCM sensor was stable across many successive replicate samples, and the method required less than 10 min, including the AChE extraction and analysis steps. This method is a rapid and convenient means for the quantification of AChE in biological samples and may be applicable for distinguishing the ventral and dorsal roots during surgical operations.

## Introduction

Peripheral nerve injury is very common both in wartime due to firearm injuries and in peacetime due to other activities. End-to-end neurorrhaphy is the treatment of choice to repair neurotmesis in clinical settings. However, in many cases nerve regeneration and functional recovery are not robust partially due to the lack of a methodology for determining the organization of the peripheral nerves. For example, if the motor and sensory tracts of the peripheral nerves are not coapted correctly during neuroanastomosis, the regenerated motor fibers will not grow onto their corresponding terminals, and the sensory fibers will fail to grow into the spinal cord, leading to the loss of sensory and motor function. Thus, the rapid and accurate identification of the motor and the sensory fibers remains one of the most challenging problems in neurosurgery.

Various methods have been proposed to detect and examine the nerve tracts, including anatomic [1], thiocholinergic [2], electrophysiological [3], radioisotopic [4], histochemical [5], and immunohistochemical [6] methods. However, these procedures often require several days to obtain results.

Several methods have been developed to rapidly identify nerve fascicles, including electrochemical methods [7], near-infrared diffuse reflectance spectroscopy [8], and Raman spectroscopy [9]. Although these methods achieve rapid identification, they are often plagued by poor selectivity and specificity of the sensing layers as well as low sensitivity (e.g., poor single-to-noise ratios), which often originate from the nonspecific adsorption of proteins and other biomolecules.

Recent advances in bioengineering have led to the development of trace detection methods for biologically active substances, such as proteins, nucleic acids, enzyme-substrates and receptor-ligand compounds. Piezoelectric immunosensors, based on quartz crystal microbalances (QCM), are specifically manufactured quartz plates with fundamental resonance frequencies of 5–30 MHz [10]. Changes in the mass of the material on the surface will alter the resonance frequency of the crystal [11], and a linear relationship exists between the deposited mass and the frequency response of the quartz crystal. This characteristic of QCM has been exploited in the development of bioanalytical tools on a 10^−9^ g scale [12]. QCM can be used to perform label-free detection of ligands, proteins and nucleic acids [11], [13], are useful for on-site monitoring and are easily used for rapid real-time multi-sample analysis [12], [14]. Therefore, QCM-based immunoassays have been developed for application in various fields [15]–[17].

Acetylcholinesterase (AChE) is found in many types of conducting tissue [18]. The levels of AChE in motor fibers are markedly higher than in sensory fibers [19]. Currently, certain AChE-based QCM devices are capable of detecting organophosphorus in agricultural products [20], [21]. An AChE antibody-based biosensing system for the rapid quantification of AChE using this same approach will be useful for rapidly detecting AChE in peripheral nerves. Although many methods are available for assaying AChE, a rapid and sensitive method remains to be developed. The objective of this study was to develop a rapid and convenient method for distinguishing between motor and sensory fibers using differences in AChE contents detected via a real-time antibody-based QCM assay. This method may be useful for distinguishing between the ventral and dorsal roots during surgical operations. Spinal ventral and dorsal roots were used in this study as examples of motor and sensory fibers.

## Materials and Methods

### Animals

A total of 10 adult beagles, weighing 7–12 kg (8.4±1.5 kg), were provided by the animal experiment center of Nanjing Medical University. This study was performed in strict accordance with the recommendations in the Guide for the Care and Use of Laboratory Animals formulated by the Ministry of Science and Technology of China. The protocol was approved by the Committee on the Ethics of Animal Experiments of Nanjing Medical University (Permit Number: 20110713). All surgery was performed under sodium pentobarbital anesthesia, and all efforts were made to minimize suffering.

### Drugs and Device

**Figure 1:**
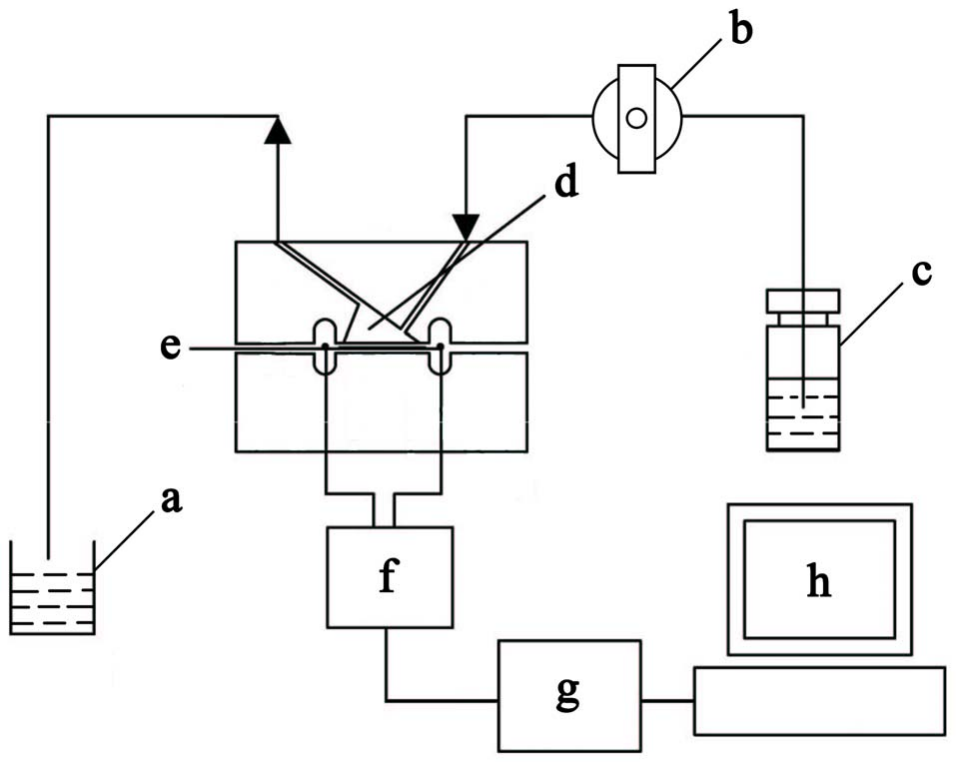
Schematic diagram of the QCM system. a, Disposal bottle; b, Pump; c, Sample cell; d, Reaction cell; e, Gold surface of QCM device; f, Oscillation circuit; g, Frequency counter; h, PC and operating software.

Phosphate-buffered saline (PBS, 0.01 M, pH 7.4) containing 0.154 M NaCl was obtained from Sigma-Aldrich (St. Louis, MO, USA). Deionized water (18.2 MΩ*cm) was produced using a Millipore-Milli-Q system (Bedford, MA, USA). The monoclonal antibody to AChE was obtained from Abgent (San Diego, CA, USA). The rabbit anti-NF200 polyclonal antibody (Wuhan boster, China), glutaraldehyde, glycine, human AChE recombinant and bovine serum albumin (BSA) were purchased from Sigma. The Alexa fluor 568 goat anti-mouse IgG and Alexa fluor 488 goat anti-rabbit IgG were purchased from Invitrogen (CA, USA). A 9-MH_Z_ AT cut piezoelectric quartz crystal slab (ANT Technology Co. Ltd., Taipei, Republic of China) with a layer of gold electrode (0.091 cm^2^; detection limit of the QCM instrument in liquid = 0.1 Hz) was used as the transducer in our experiment. The flow injection and continuous frequency variation recording were performed using an Affinity Detection System (ADS; ANT Technology Co. Ltd. Taipei, Republic of China). The system has five main components: an electronic oscillation circuit, a frequency counter, a piezoelectric quartz fixed biosensor molecule (p-chip), a single-loop flowsystem and a computer used to record and analyze the frequency change (ΔF) curve in real time (Figure 1). The sensor unit had the following properties: resolution (0.1 Hz), sampling period (1 s), frequency range (2–16 MHz), temperature range (4–60°C) and voltage (220 V, 50–60 Hz). The reaction cell was a sensor signal channel with a 30 µl reaction cell volume. The single-loop flowsystem consisted of a temperature controller, sample tubes, pipelines and a tubing pump with a flow rate of 10–200 µl/min and a sample loop volume of 100 µl. In our experiment, the gold electrode surface of the QCM was modified with polyethylene imine (PEI) using the plasma method by the manufacturer.

### Surgical Procedures

Ketamine (15 mg/kg), droperidol (5 mg), and atropine (0.5 mg) were used to induce anesthesia, and the animals were sacrificed using an intravenous injection of air. The spinal canal was exposed via a median incision in the lumbosacral region with the dog lying prone, and both the ventral and dorsal roots were then excised for the experiment (L1–S2). The root specimens were washed with PBS and then immediately frozen with liquid nitrogen.

### QCM System Preparation

The real-time frequency shift of the QCM device was recorded by the computer. The activation and immobilization of the QCM device were performed with glutaraldehyde and an AChE antibody, as previously described with minor modifications. In brief, 2.5% glutaraldehyde was injected into the reaction cell to activate the chip of the QCM device. The AChE antibody (100 µg/ml) was then added to the reaction cell to be immobilized on the chip. The remaining active carboxaldehyde groups were blocked by an injection of 1 M glycine solution into the system. Following the injection, the AChE antibody-modified sensor chip was ready to detect samples. The chip could be stored in PBS at 4°C for 1 month.

### Procedure for AChE Detection

The ventral and dorsal roots were cut into 1- to 2-mm-long segments. The AChE from the cross-sectional surface of the roots was dissolved in 250 µl PBS (0.01 M) for 1 min. The solutions were cleared by centrifugation for 3 min at 12,000×*g*. The supernatant (200 µl) was collected for AChE detection. The gold-QCM device was exposed to the sample flow containing solutions of the nerve roots and 5% BSA. The reaction between the AChE antibody and AChE in the QCM device occurred at a flow rate of 70 µl/min. The frequency shift during the reaction was recorded using real-time observations.

The ΔF value observed via real-time continuous reading was reported as the difference between the final value and the value prior to the antigen-antibody reaction or immobilization. The mass of immobilized glutaraldehyde as well as the mass of the AChE antibody and AChE protein on the QCM device was calculated using Sauerbrey’s equation [22]:
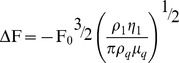
where ΔF is the measured frequency shift, F_0_ is the resonant frequency of the unloaded crystal, *ρ*_l_ is the density of the liquid in contact with the crystal, *η*_l_ is the viscosity of the liquid in contact with the crystal, *ρ*_q_ is the density of quartz (2.648 g cm^−3^) and *µ*_q_ is the shear modulus of quartz (2.947×10^11^ g cm^−1^ s^−2^). A frequency change of 1 Hz corresponds to a mass change of 0.883 ng.

### Enzyme-linked Immunosorbent Assay (ELISA)

The AChE levels in the ventral and dorsal roots were quantified using solid-phase ELISA. Tissues (1 to 2 mm long) were washed in 200 µl PBS, centrifuged at 12,000 × *g* for 5 min at 4°C and incubated on plates at 4°C overnight. The AChE antibody was added to the plates, which were then incubated for 1 h at 37°C. Goat anti mouse–peroxidase conjugate was added to quantify the binding of the secondary antibody. After chromogenic by 3,3′,5,5′-Tetramethylbenzidine (TMB) and stopping the reaction by the addition of citric acid, the absorbance was measured at 450 nm.

### Double Immunofluorescent Staining

Double immunofluorescent staining for AChE and NF200, a specific marker of neurofilaments, was used to determine the regional expression of AChE in the ventral and dorsal roots. Staining was performed according to standard procedures. Briefly, the sections were deparaffinized in xylene, cleared using a graded ethanol series in phosphate buffered saline (PBS) and then placed in 10 mmol/l citrate buffer (pH 6.0) for 15 min at 100°C for antigen retrieval. The sections were incubated with a mouse anti-AChE monoclonal antibody overnight at 4°C and were then conjugated using Alexa fluor 568 goat anti-mouse IgG. After rinsing the sections in Tris-buffered saline (TBS), rabbit anti-NF200 polyclonal antibody and Alexa fluor 488 goat anti-rabbit IgG were applied. The sections were placed in Gel Mount aqueous mounting medium under a cover glass and were examined using an Olympus BX51 microscope. Controls were obtained by omitting the primary or secondary antibodies. No staining was observed in the negative control conditions. Images were taken at 200× magnification.

### Data Analysis

The experimental data were analyzed using the P-Sensor software of the ADS system in real time. Each experiment was repeated three times. All data are presented as the mean ± standard deviation (S.D.). Differences between the groups were evaluated using a two-tailed Student’s t-test. P-values less than 0.05 were considered to represent statistically significant differences.

## Results

### Detection of Pure AChE using the QCM Device and ELISA

**Figure 2:**
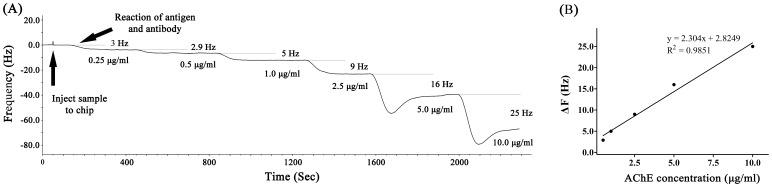
Detection of AChE using the QCM system. (A) Detection of various concentrations of AChE in solution using the QCM system. The frequency shift increased as the concentration of AChE increased. (B) Frequency shifts of the QCM immunosensor as a function of the concentration of AChE in PBS. The concentration of the AChE antibody was 100 µg/ml. A linear correlation between the frequency change and the concentration of AChE was observed between 0.5 to 10 µg/ml.

**Figure 3:**
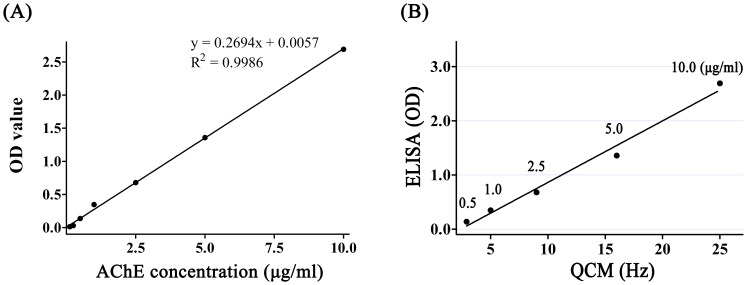
Detection of AChE using ELISA. (A) The OD value increased with increasing concentrations of AChE. A linear correlation was observed between the OD values and the concentrations of AChE from 0.125 to 10 µg/ml. (B) The correlation between QCM and ELISA was analyzed using SPSS. The correlation coefficient was 0.9924 (P<0.05), suggesting a strong correlation between the QCM and ELISA results.

Various concentrations of AChE solution (0.125–10.0 µg/ml) were tested using the antibody-based QCM immunosensor and ELISA. As shown in Figure 2A, the recorded frequency shift increased following the addition of AChE. The detection time for each measured solution was 6 min. In our experiments, the AChE solution was immobilized on the chip surface by the affinity reaction using AChE antibody. Figure 2 showed a typical response for the QCM frequency shift measured in situ for the reactions. As shown in Figure 2A, 0.5 µg/ml AChE is introduced into the QCM causes an initial rapid frequency decrease due to mass loading and the saturation frequency shift is 2.9 Hz. The mass loading at saturation is 2.56 ng as calculated using the Sauerbrey equation. The frequency shift increases to 5 Hz with AChE concentration increasing to 1.0 µg/ml and the mass loading is 4.14 ng. The frequency shift for AChE concentration showed a 1∶1 correspondence with mass loading. In this case, a linear correlation was found between the frequency change and the concentration of AChE from 0.5 µg/ml to 10 µg/ml (Figure 2B). The linear relationship between the OD values and the concentrations of AChE protein from 0.25 µg/ml to 10 µg/ml is shown in Figure 3A. Moreover, both methods had similar dynamic ranges. A typical ELISA assay experiment requires more than 2 h, whereas the QCM-based method requires only 10 min to obtain the results. Thus, the QCM-based method may be more useful for rapid estimates of AChE levels.

Various concentrations of the AChE standard solution were tested using ELISA. The OD values increased with increasing concentrations of AChE (Figure 3A). The correlation between QCM and ELISA was analyzed using SPSS. The correlation coefficient was 0.9924 (P<0.05), suggesting a strong correlation between the QCM and ELISA results (Figure 3B).

### Detection of AChE in Ventral and Dorsal Spinal Roots

**Figure 4:**
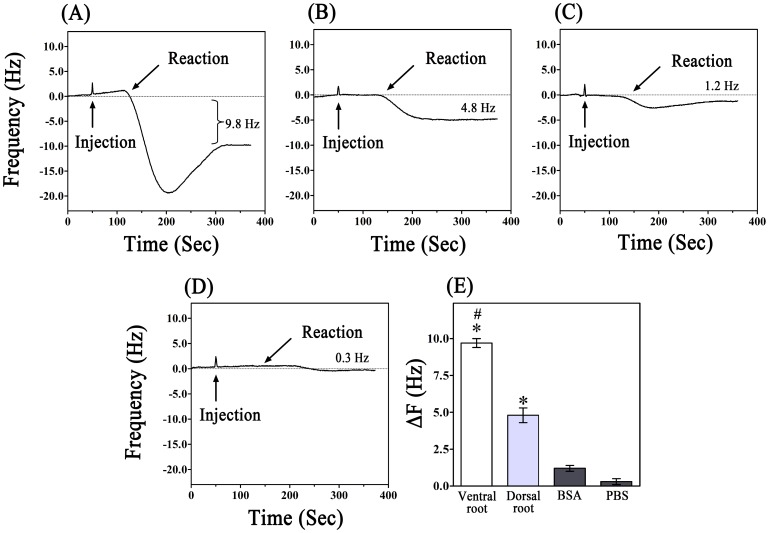
Detection of solutions of the ventral and dorsal roots using the QCM system. (A) The detection process for the ventral root solution. A 9.8-Hz decrease in the frequency was induced by the ventral root solution. (B) AChE in the dorsal root solution was also detectable using the QCM device. A 4.8-Hz frequency shift was induced by the dorsal root solution. (C), (D) The BSA and PBS showed only a relative small reaction with the antibody on the chip. The frequency shifts produced by the BSA and PBS were 1.2 and 0.2 Hz, respectively. (E) The frequency change (ΔF) after the antigen-antibody reaction was measured and calculated. ^#^P<0.05, vs. dorsal root, BSA and PBS. *P<0.05, vs. BSA and PBS.

In the current experiments, the formation of immuno-complexes was monitored in real time based on ΔF. As shown in Figures 4A and B, AChE protein in both the ventral and dorsal roots was detectable in the QCM system. The detection process was completed within approximately 6 min. PBS and BSA were also assayed using the QCM device (Figures 4C and D). The assay of the dorsal roots resulted in a 4.8±0.5 Hz frequency shift, whereas the assay of the ventral roots resulted in a 9.7±0.3 Hz frequency shift. The concentrations of AChE in the ventral and dorsal roots were 2.98 and 0.9 µg/ml, respectively, according to the corresponding formula determined using pure AChE protein. The ΔF results from the PBS and BSA assays were 0.3±0.2 and 1.2±0.2 Hz, respectively. These data indicated that the AChE antibody was able to specifically bind to the complementary antigen. The ΔF induced by the ventral roots was greater than that induced by the dorsal roots, indicating that the AChE content in the ventral roots was significantly higher than in the dorsal roots (p<0.05). The ΔF values induced by PBS and BSA were notably lower than those of the ventral and dorsal roots (Figure 4E). The results indicated that AChE could be distinguished from blank buffers and another protein. To further confirm our data, the samples were also analyzed using ELISA; the concentrations of AChE in solutions of the ventral and dorsal roots were determined to be 2.80 and 1.02 µg/ml, respectively.

### Stability of the QCM System

**Figure 5:**
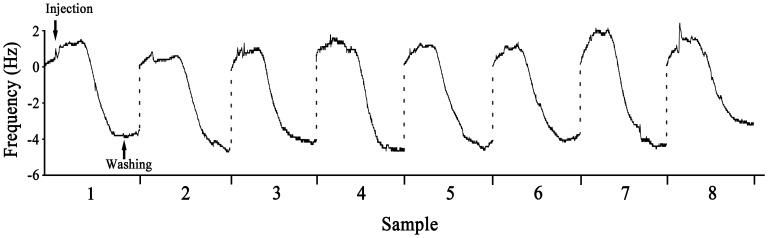
Stability of the QCM system. Eight identical samples were consecutively detected after immobilization of the AChE antibody. The frequency shift was recorded using ANT software. Each sample induced an approximately 5 Hz shift in the frequency. The variance was 0.099, and the standard deviation was 0.315.

The stability of the QCM system was assessed using eight identical samples (AChE protein, 1.0 µg/ml) for continuous detection on the same crystal after immobilization of the AChE antibody (Figure 5). The frequency shifts caused by the AChE protein were recorded using the ANT software. Each sample induced an approximately 5 Hz frequency change. The variance was 0.099, and the standard deviation was 0.315, which was indicative of the stability of the instrument after repeated sample measurements. These results also indicated that the irreversible binding of AChE on the antibody-coated sensor was negligible, thus offering the opportunity for analysis of multiple clinical samples without changes to the system. Moreover, we noticed that there was no loss of sensitivity when the AChE antibody-modified sensor was stored at 4°C for up to 1 month.

### Immunohistochemical Staining of Ventral and Dorsal Roots

**Figure 6:**
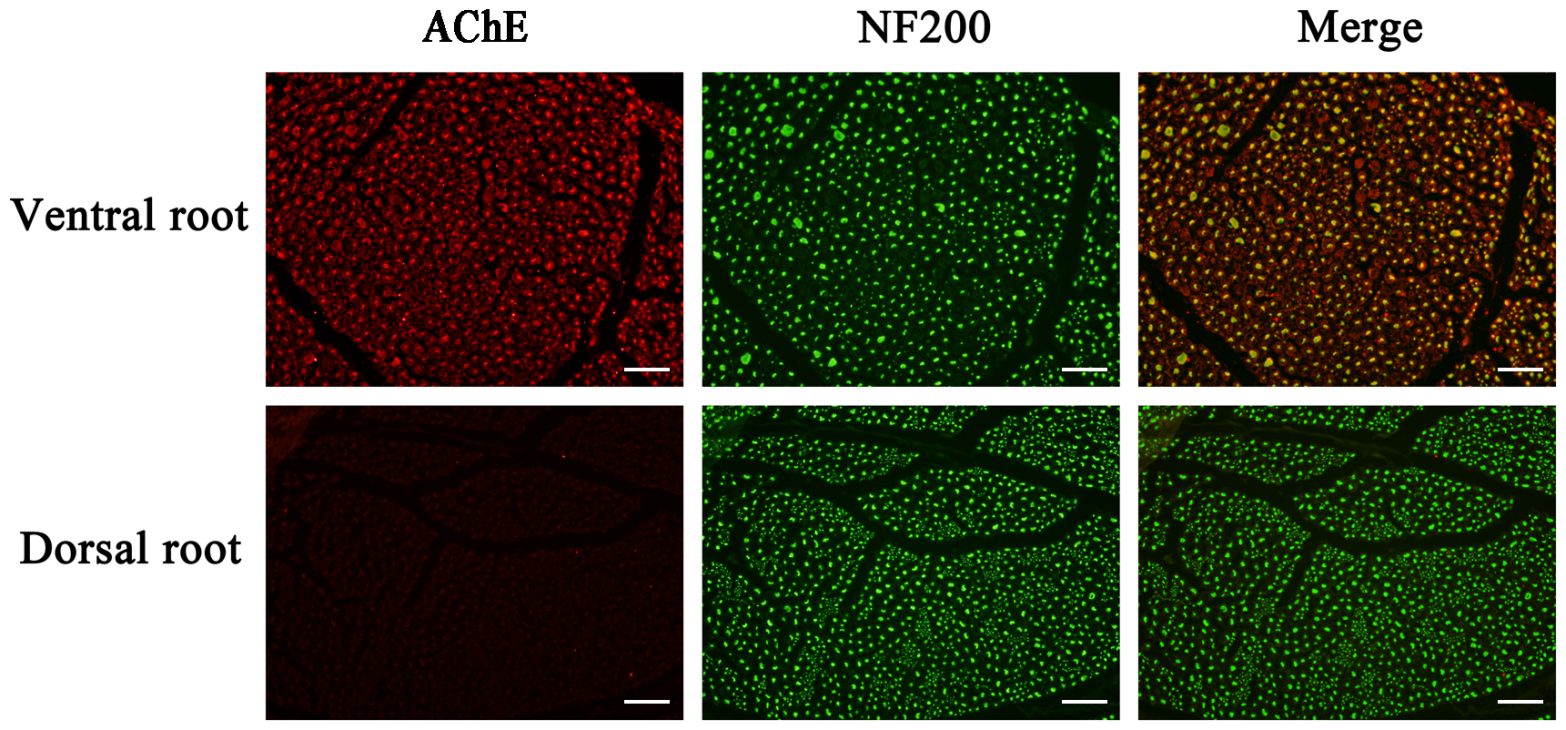
Differences in the axonal distribution of AChE between the ventral and dorsal roots. The axons of the neurons are labeled with anti-NF200 (green). The AChE protein (red) was detected in the ventral roots, but was detected only at low levels in the dorsal roots. Merged images of AChE and NF200 staining are shown on the right. Scale bar, 50 µm.

The ventral and dorsal roots were sectioned and subjected to immunohistochemical examination after QCM detection. As shown in Figure 6, the neurons, indicated by green fluorescence, were labeled with NF200 in both the ventral and dorsal roots. AChE was not detectable in the neurons of the dorsal roots. However, we observed strong positive staining of AChE in the neurons and surrounding myelin sheathes of the ventral roots. This finding supported the concept that AChE is mainly present in spinal ventral roots and that the concentration of AChE in the ventral roots is greater than in the dorsal roots.

## Discussion

The ventral root contains primarily motor nerves, and the dorsal root contains primarily sensory nerves. The main difference between the motor and sensory fibers is that acetylcholine is the neurotransmitter in the motor fibers, whereas substance P is the neurotransmitter in the sensory fibers [3], [23]. The AChE content is notably greater in the ventral roots that consist of motor fibers than in the dorsal roots that consist of sensory fibers [19], [24]. In this study, we observed stronger positive AChE staining in the neurons and surrounding myelin sheaths of the ventral roots than the dorsal roots. Therefore, the detection of AChE could be a useful method for delineating peripheral nerves. Several methods can be used to detect the AChE, such as a colorimetirc test, AChE staining, a radioisotopic method or immunohistochemical staining. The colorimetric test is rapid but suffers from interference by butyrylcholinesterase (BChE) [25]. AChE staining can be used to successfully delineate ventral and dorsal roots, but this approach requires at least 1 h to ensure correct diffferentiation [2], [26]. The radioisotopic method requires 3 h or more to identify motor and sensory nerves, and, although it may be more sensitive, it is more expensive than staining methods. The bright blue-labeled monoclonal Blue-SAB specifically reacts with the cell body and may be used to identify sensory fibers of nerve trunks using immunohistochemical techniques. Unfortunately, these sophisticated methods are costly and time consuming.

In the present study, we used QCM to develop a rapid, highly sensitive antibody-based piezoimmunosensor assay for detecting AChE in solution. This method, and other similar assays, can potentially be used to quickly identify proteins, expediently monitor antigen or antibody attachment to the quartz surface and ensure antibody-antigen specific recognition [27]. The frequency change of the QCM corresponds to the level of AChE in a concentration-dependent manner (0.5–10 µg/ml). ELISA assays confirmed the results of the QCM assay with a correlation coefficient of 0.9924 (P<0.05) between the methods. These results indicated that the QCM was comparable to the ELISA in terms of sensitivity for AChE detection (0.5–10 µg/ml vs 0.25–10 µg/ml) and sample volume (200 µl vs 200 µl). The differences in the levels of AChE in the solutions of the ventral and dorsal roots were detected using QCM. According to the results of the QCM, the concentration of AChE in the solutions of the ventral roots was 2.98 µg/ml, and the concentration in the dorsal roots was 0.90 µg/ml. In comparison, according to the ELISA results, the contents of AChE in the solutions of the ventral and dorsal roots were 2.80 and 1.02µg/ml, respectively. These results were also demonstrated using immunohistochemical staining. Our observations are consistent with those of previous studies [5], [24]. The significantly higher AChE contents detected in the ventral roots than in the dorsal roots could be used to verify the identity of the nerve roots. BSA and PBS did not induce a detectable ΔF, indicating that BSA and PBS did not bind to the surface of the QCM.

The QCM is an ultra-sensitive weighing device, by which mass loading below 1 ng/cm^2^ can be determined [28], [29]. The shift in resonance frequency is linearly related to the loading mass by the Sauerbrey equation. But in the case of antibody/antigen binding detection, results of the experimental frequency decreases differ from those of the theoretically calculated values. For example, when 200 µl solution of AChE with the 1 µg/ml concentration was injected into the sample loop in the QCM used in the present study, the total mass of 20 ng AChE should be loaded on the surface of the gold electrode (The volume of sample loop is 100µl, and the radius of gold electrode is 0.175 cm and the reaction chip is 0.4 cm; thus the gold electrode area is 1/5 of reaction chip area, resulting in the loading mass of AChE to be 0.1 ml × 1 µg/ml × 1/5 = 20 ng). According to Sauerbrey equation, the 22.65 Hz frequency shift should be detected, but the experimental data was found to be only 5 Hz (Figure 2B), about 22% of the theoretical data. The results in AChE solutions with various concentrations were similar to this result, with the ratio of experimental data/theoretical data being about 11–26% (data not shown). Our results were consistent with previously published data in Babacan’s report [30]. The Sauerbrey equation is good for thin and rigid layers. In our system the mass of AChE attached to the surface was dependent on various factors, such as the effectiveness of the antibody binding to the protein, the failure of proteins to form rigid and firm layers due to the delicate nature, and the change of density and viscosity of the liquid by the injected protein solutions. Therefore, the difference between experimental and theoretical values was not surprising, though the protein mass should be cautiously predicted on the QCM using Sauerbrey equation. However, the frequency shift in this study gave a linear correlation between the frequency change and concentration of AChE (from 0.5 µg/ml to 10 µg/ml) (Figure 2B). These results were confirmed in a simultaneously conducted study by ELISA. It was shown that QCM as a biosensor in the liquid phase could provide a useful measurement method to detect proteins in the solution [29], [31], [32].

Using our procedure, the total time required for chip preparation to determining the ΔF of the first sample was approximately 1.5 h. The extraction and detection of AChE (identification of nerve roots) could be achieved within 10 min using a pre-modified chip. Moreover, the ΔF response was stable when tested with repeated samples of the same concentration of AChE solution. All of these results suggest that our method for delineating motor and sensory fibers using AChE concentration, as detected by a real-time antibody-based QCM assay, can potentially be used in neuroanastomosis. AChE is also found in the membranes of red blood cells, where it comprises the Yt blood group antigen [33]. It is necessary to wash and remove any blood from the nerve samples before detection. Alternatively, a specific monoclonal antibody that recognizes only AChE in nerve fibers could be developed.

In conclusion, an antibody based QCM assay method was developed for the quick, label-free, sensitive, convenient and specific detection of AChE. The spinal nerve roots could be identified according to their AChE content. Detection could be completed within 6 min. The results indicated that our method has great potential as an alternative method for the identification of nerve roots during surgery and for the identification of other branches of peripheral nerves, such as the ulnar, radial and peroneal nerves, to aid in the correct anastomosis of nerve stumps and to improve nerve regeneration and functional recovery.
